# Myocardial Infarction Due to Paradoxical Thromboembolism Originating From Distal Lower Extremity Deep Vein Thrombosis (LEDVT)

**DOI:** 10.7759/cureus.34592

**Published:** 2023-02-03

**Authors:** Meagan Josephs, D. Juliet Benonaih-Jumbo, Thanushiya Jeyakanthan

**Affiliations:** 1 Internal Medicine, American University of the Caribbean School of Medicine, Cupecoy, SXM; 2 Internal Medicine, Norwalk Hospital, Yale University, Norwalk, USA

**Keywords:** echocardiogram, myocardial infarction, patent foramen ovale, thrombus, paradoxical emboli

## Abstract

Paradoxical embolism (PDE) originates in the venous system and ends up in the arterial circulation via cardiac or pulmonary shunts. Cases of PDE from venous thrombosis resulting in acute myocardial infarctions (MIs) are seldom reported in the literature. Diagnoses can often be missed if further workups are not pursued in patients without any underlying risk factors for coronary artery disease (CAD). Here, we report a case of a paradoxical embolus that crossed the patent foramen ovale (PFO), causing ST-elevation MI (STEMI) from an embolized venous thrombus originating in the left distal posterior tibial vein.

## Introduction

Venous thromboembolism (VTE) is a common phenomenon encountered in healthcare practice as deep vein thrombosis (DVT) and pulmonary embolism (PE). Usually, DVT and PE originate and can embolize to other locations in the venous system. Interestingly, when a venous clot ends up in the arterial circulation, it is then called a paradoxical embolism (PDE) [1]. Most VTE originates in the proximal femoral vein. Lower extremity DVT (LEDVT) accounts for only 20% to 30% of DVT as calf veins are not routinely scanned and imaging studies are less sensitive in this region [2]. However, in the presence of an intracardiac or pulmonary shunt, LEDVT has the potential to migrate into the arterial circulation and cause major ischemic events [3]. Although congenital heart defects such as patent foramen ovale (PFO) affect as much as 34% of the adult population, they are often found incidentally and very rarely result in PDE [4]. PDE accounts for <2% of all arterial emboli [5]. PDE is often reported in patients aged under 55 years [6]. PDEs most commonly result in cerebral vascular accidents (CVAs), known as cryptogenic strokes, which may cause neurologic deficits such as unilateral deficits or problems with speech or vision. However, patients may present with an array of symptoms, including abdominal pain, chest pain, or limb ischemia depending on the location of thrombus embolization [3,7].

Myocardial infarction (MI) from PDE is a rare diagnosis that requires a high degree of clinical suspicion and thorough workup as it is a diagnosis of exclusion. Other causes of MI, including but not limited to coronary artery disease (CAD), vasospasm, valvular or conduction defects such as atrial fibrillation (AF), and less commonly, collagen defects and vasculitides, must be first ruled out [8]. PFOs are common, although usually asymptomatic; rarely, transient increases in intrathoracic pressure can change blood flow, so it moves from right to left. The resultant PDE very rarely occludes a coronary artery to cause an acute MI [3,7]. Presumed diagnosis is typically made with a transthoracic echocardiogram (TTE), although visualization and resolution will not always be clear. Transesophageal echocardiogram (TEE) with Doppler flow interrogation is the gold standard as it allows for direct characterization of the PFO [9]. It is imperative in identifying the anatomical defect that leads to PDA as these patients are at risk of life-threatening future embolic events [5].

The combination of low prevalence and other common causes for cardiac arrest makes MI from PDE a unique and difficult diagnosis to make on initial presentation. Our report presents a case of a PDE causing ST-elevation MI (STEMI) secondary to a thrombus originating from the left distal posterior tibial vein.

This study was previously submitted as an abstract at the 2021 American College of Prosthodontists (ACP) Connecticut Chapter on October 15, 2021.

## Case presentation

A 31-year-old physically fit Latino male with no prior medical history was brought to the emergency department (ED) with acute substernal chest pain radiating to the left arm that woke him from sleep. The patient had a distant history of a *mitral valve not closing properly* that required prolonged monitoring but no intervention. There is no family cardiac history. He is a nonsmoker. The patient reported recent swelling of his legs following a long bike ride but continued to cycle 30 to 60 miles per day.

**Figure 1:**
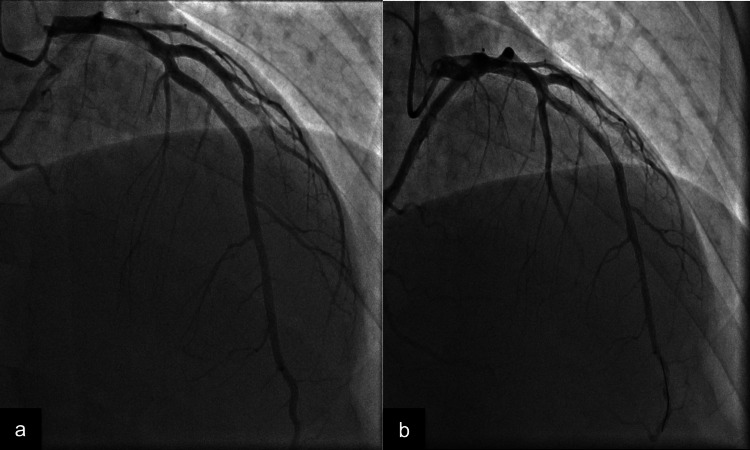
(a) A 16 mm, 80% de novo occlusion of LAD; (b) captured after drug-eluting stent placement with a brisk return of blood flow (TIMI grade 3). LAD, left anterior descending; TIMI, thrombolysis in myocardial infarction

On arrival, he was diaphoretic, afebrile, and normotensive, with a heart rate (HR) of 59 beats per minute, respiratory rate (RR) of 16 breaths per minute, and saturating well on room air. Echocardiogram (EKG) showed normal sinus rhythm with left axis deviation and diffuse ST elevation in the anterolateral leads. Chest X-ray was unremarkable. Cardiac biomarkers were elevated (troponin T 52-2,323 ng/L (normal <0.01 ng/L) and creatinine kinase 1061 U/L). The patient was anticoagulated with heparin 5,000 IU and Brilinta (ticagrelor, AstraZeneca, Cambridge, UK) 180 mg then emergently transferred to the catheter lab. A coronary angiogram revealed an 80% de novo occlusion of the proximal left anterior descending (LAD; Figure 1a) artery. Multiple thrombi were aspirated, and a drug-eluting stent was placed with a brisk return of blood flow (Figure 1b). The remaining coronary arteries were free of arteriosclerotic disease.

**Figure 2:**
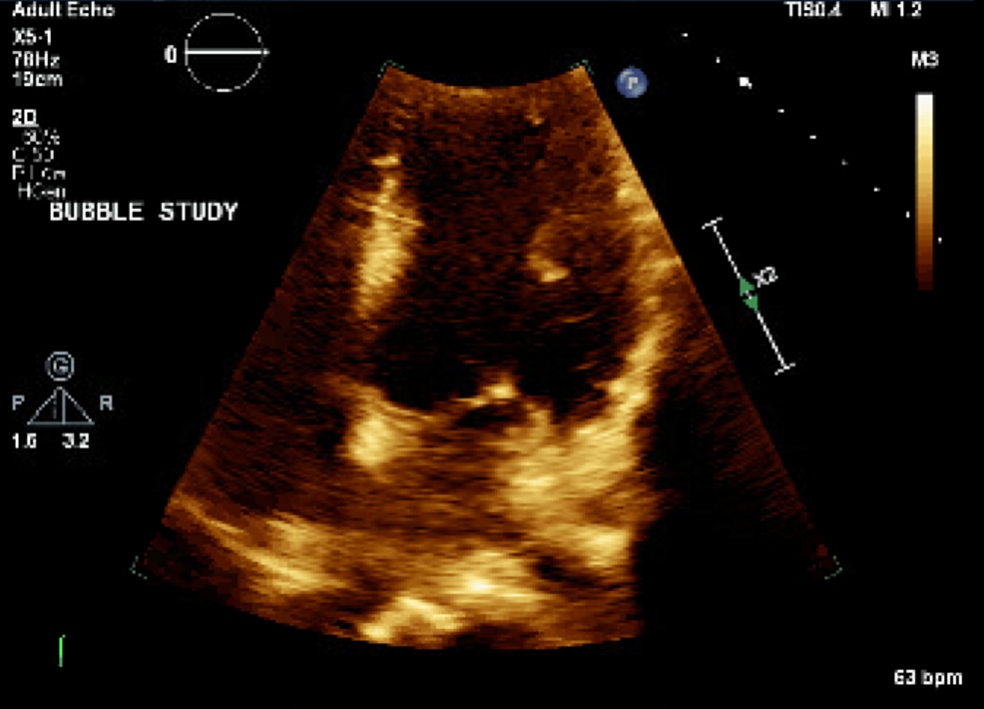
Transthoracic echocardiogram with agitated saline showed evidence of a PFO, and transesophageal echocardiogram with a bubble study confirmed the presence of a PFO measuring 2 to 3 mm. PFO, patent foramen ovale

**Figure 3:**
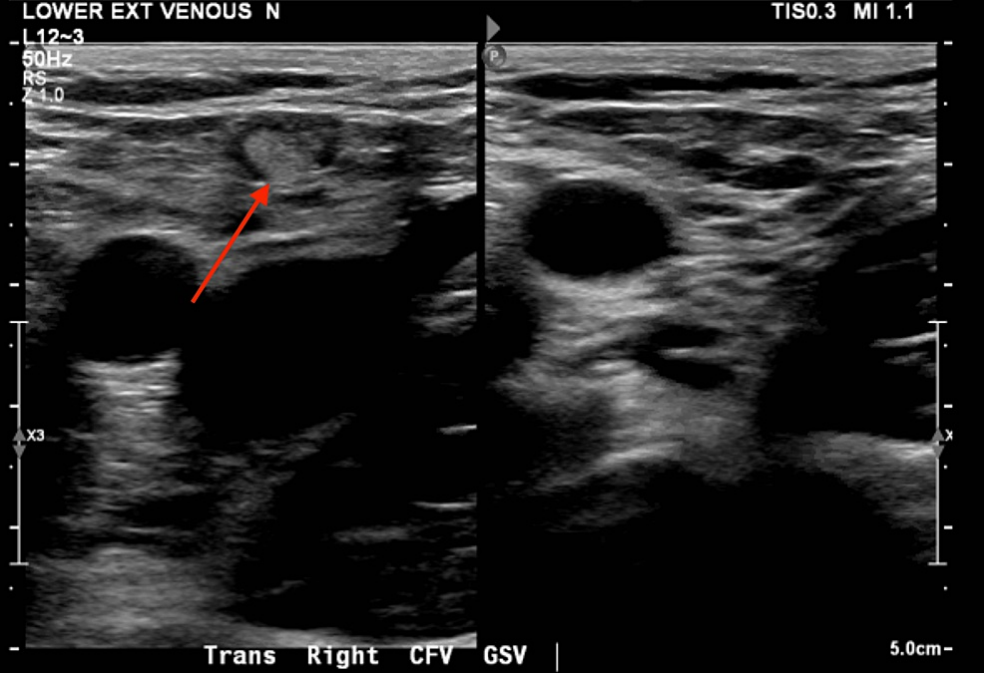
Bilateral lower extremity venous duplex ultrasound performed revealed occlusive thrombosis of the left distal posterior tibial vein.

The patient was transferred to the ICU for further monitoring and diagnostic workups. TTE with saline agitation showed evidence of a right-to-left shunt (Figure 2). This was later confirmed by a TEE with a bubble study that showed a small PFO with a bidirectional shunt measuring 2 to 3 mm. A bilateral lower extremity venous duplex ultrasound revealed occlusive thrombosis of the left distal posterior tibial vein (Figure 3). The lipid panel was within the normal range. Given the radiologic findings and no evidence or risk factors for CAD, it was suspected that a PDE resulted in MI. Repeat EKG showed resolution of ST segment changes and troponin down trended. No further chest pain was noted since catheterization.

Upon discharge, the patient was initiated on one month of dual antiplatelet therapy (Aspirin and Plavix), lisinopril, and high-intensity statin per the cardiologist’s recommendations. The patient was discharged with therapeutic apixaban with plans to continue prophylactic dosage for six months. Extensive hypercoagulability workup in the outpatient setting was inconclusive. The patient has since undergone PFO closure to prevent future events.

## Discussion

The first account of PDE was detailed by Cohnheim in 1877 when he described the path of an embolus through a septal defect in the heart [5]. However, Zahn coined the term *PDE* in 1885 to describe an embolus passing from the venous to the arterial system through intracardiac communication. Four criteria must be met to diagnose a PDE, including a venous embolic source, a right-to-left shunt, a pressure gradient across the shunt, and an arterial embolism [7,10].

Emboli may also be composed of fat, air, or amniotic fluid though most are thrombi formed in the venous circulation [3]. DVTs have an annual incidence as high as 67 per 100,000 in the general population with 70% to 80% of those detected in the proximal veins.

LEDVT makes up a small proportion of diagnosed DVTs as calf veins are not routinely scanned and imaging studies are less sensitive in this region [2]. In the presence of intracardiac or pulmonary shunts, thromboembolisms have the potential to cause major ischemic events; therefore, treatment is highly recommended.

PFO affects 20% to 34% of the adult population although most patients are asymptomatic [11]. PFO occurs due to failed fusion of the septum primum and septum secundum in the fourth week of gestation [9]. Normally, PFO remains closed due to the pressure gradient between the left and right atrium. However, transient increases in right atrial pressure, including coughing, squatting, or defecating, can reverse the direction of blood flow and cause emboli to pass into the arterial system [3,7]. PFO is often found incidentally, but it is also the most common shunt associated with PDE [4,11].

PDE may also occur in association with atrial septal defects (ASDs), ventricular septal defects (VSDs), or pulmonary arteriovenous malformations (PAVMs) [7]. In contrast to PFO, patients with VSD causing Eisenmenger syndrome or PAVM have persistent right-to-left shunts that can result in PDE [3,12].

Less than 2% of systemic arterial emboli are paradoxical, and most PDEs affect the cerebral vasculature to cause cerebrovascular events; in fact, up to 77% of cryptogenic strokes have been associated with PFO [5,6,8].

PDE may also embolize to the peripheral, renal, or mesenteric arteries or less commonly to the coronary arteries [10]. MI comprises only 10% to 15% of PDEs [5]. Accordingly, MI from PDE has been infrequently reported in the literature. Singh et al. reported a presumed case of PDE causing recurrent MI in a 68-year-old female with a history of paroxysmal AF [13]. However, rates of MI are higher in patients with AF even in the presence of stable atherosclerosis and the use of anticoagulation [14]. Similarly, Jamiel et al. reported the case of a 16-year-old boy who developed a thrombus in the left circumflex artery following a Glenn operation for the closure of ASD [4]. Unlike our case, these cases highlight patients with some previous cardiac history.

Kleber et al. determined that patients with presumed MI from PDE are significantly younger than the general population who are affected by MI (43.0 ± 12.0 vs. 63.2 ± 12.7 years) [8]. However, PDE should always be considered as an etiology when patients with no clear risk factors present with an MI [3]. Other considerations may include CAD, vasospasm, valvular or conduction defects such as AF, and less commonly, collagen defects and vasculitides that must be ruled out [8].

A diagnosis of an acute MI can be made with EKG and laboratory tests, including troponin and creatine kinase [8]. MI from PDE is a diagnosis of exclusion that can be presumed with further workup. Coronary angiography or coronary magnetic resonance imaging (MRI) will show an acute occlusion in otherwise intact vessels [3]. Initial investigation of an intracardiac shunt involves a TTE with Doppler imaging although sensitivity is low (87%) when imaging small shunts, so it may need to be followed by a more invasive TEE with a bubble study. The presence of an atrial shunt can be presumed if bubbles are located in the left atrium within five beats; external abdominal compression to reverse shunt flow further improves visualization [9]. In the presence of a shunt, further studies, including computed tomography (CT) or MRI may be warranted to evaluate for thromboembolic complications [10]. Additionally, patients may require lower extremity ultrasound and hypercoagulability tests, including activated prothrombotic time, prothrombin time, protein C and S, antiphospholipid antibodies, and fibrinogen to look for the etiology of the embolus [3,9].

Aspiration embolectomy with or without stenting is the primary intervention for the management of MI from all causes [5]. Following the procedure, patients should be started on antiplatelet therapy and anticoagulation for DVT or pulmonary embolism [4]. Aspirin alone is insufficient for secondary prevention, but some patients may require lifelong antiplatelet therapy [3,7]. In addition, percutaneous closure of PFO has been shown to significantly reduce the risk of recurrent stroke compared to medical management without increasing the risk of major bleeding [15]. There is little evidence to support PFO closure to reduce the risk of recurrent MI, but this procedure should be considered to prevent recurrent ischemia [5,13]. Although a generally safe, outpatient procedure, PFO closure is not without risk; complications include vascular injury, device embolization, cardiac tamponade, and atrial arrhythmias [7,15].

## Conclusions

PDE is an uncommon cause of MI that should be considered in patients without cardiac risk factors or family cardiac history. Patients with suspected MI from PDE should be promptly diagnosed to reduce the risk of future embolic events.

MI from PDE is a diagnosis of exclusion, following the initial detection of MI indicated by elevated biomarkers and EKG changes. The presence of an atrial shunt detected by TEE with a bubble study and evidence of thrombosis by ultrasound further support the diagnosis. Embolectomy is the primary intervention. Patients should be initiated on antiplatelet and anticoagulant therapy along with surgical closure of the inciting PFO.
